# Effect of transanal drainage tube on prevention of anastomotic leakage after anterior rectal cancer surgery taking indwelling time into consideration: a systematic review and meta-analysis

**DOI:** 10.3389/fonc.2023.1307716

**Published:** 2024-01-23

**Authors:** Xinzhen Xu, Xiang Zhang, Xin Li, Ao Yu, Xiqiang Zhang, Shuohui Dong, Zitian Liu, Zhiqiang Cheng, Kexin Wang

**Affiliations:** ^1^ Department of General Surgery, Qilu Hospital of Shandong University, Jinan, China; ^2^ Department of General Surgery, Huantai Country People’s Hospital, Zibo, China

**Keywords:** anastomotic leakage, indwelling time, transanal drainage tube, anterior resection, rectal cancer

## Abstract

**Background:**

Placement of an indwelling transanal drainage tube (TDT) to prevent anastomotic leakage (AL) after anterior rectal cancer surgery has become a routine choice for surgeons in the recent years. However, the specific indwelling time of the TDT has not been explored. We performed this meta-analysis and considered the indwelling time a critical factor in re-analyzing the effectiveness of TDT placement in prevention of AL after anterior rectal cancer surgery.

**Methods:**

Randomized controlled trials (RCTs) and cohort studies which evaluated the effectiveness of TDT in prevention of AL after rectal cancer surgery and considered the indwelling time of TDT were identified using a predesigned search strategy in databases up to November 2022. This meta-analysis was performed to estimate the pooled AL rates (Overall and different AL grades) and reoperation rates at different TDT indwelling times and stoma statuses.

**Results:**

Three RCTs and 15 cohort studies including 2381 cases with TDT and 2494 cases without TDT were considered eligible for inclusion. Our meta-analysis showed that the indwelling time of TDT for ≥5-days was associated with a significant reduction (TDT vs. Non-TDT) in overall AL (OR=0.46,95% CI 0.34-0.60, *p*<0.01), grade A+B AL (OR=0.64, 95% CI 0.42-0.97, *p*=0.03), grade C AL (OR=0.35, 95% CI 0.24-0.53, *p*<0.01), overall reoperation rate (OR=0.36, 95%CI 0.24-0.53, *p*<0.01) and that in patients without a prophylactic diverting stoma (DS) (OR=0.24, 95%CI 0.14-0.41, *p*<0.01). There were no statistically significant differences in any of the abovementioned indicators (*p*>0.05) when the indwelling time of TDT was less than 5 days.

**Conclusion:**

Extending the postoperative indwelling time of TDT to 5 days may reduce the overall AL and the need for reoperation in patients without a prophylactic DS.

**Systematic review registration:**

https://www.crd.york.ac.uk/prospero/display_record.php?ID=CRD42023407451, identifier CRD42023407451.

## Introduction

Anastomotic leakage (AL) is a major complication of anterior rectal cancer surgery, and its incidence has been reported to range between 1- 30% (1, 2). The occurrence of AL could result in significantly more severe postoperative complications, higher rates of reoperations, increased hospital stay, and higher mortality. It can even affect the patients’ prognosis (3). The endoluminal pressure at the anastomotic site may be associated with AL (4). Placing a transanal drainage tube (TDT) can reduce the intraluminal pressure and help drain feces and gas at the anastomosis site (5, 6). This is likely to provide an ideal regional environment for anastomosis healing, thus reducing the incidence of AL. However, there is a controversy regarding the effectiveness of TDT placement for prevention of AL after anterior resection of rectal cancer (5, 7–13). Tamura et al. (10) analyzed 157 patients who underwent laparoscopic anterior resection with and without postoperative TDT, and reported that the AL rate was not statistically significant between the two groups (7.6% vs 10.3%, TDT vs. non-TDT). Zhao et al. (12) conducted a randomized clinical trial to assess the effect of TDTs in AL prevention after laparoscopic low anterior resection for rectal cancer in 576 patients and concluded that TDTs may not confer any benefit for AL prevention in patients who undergo laparoscopic low anterior resection for mid-low rectal cancer without preoperative radiotherapy. However, Kawada et al. (14) found that TDT significantly reduced the AL rate from 26.1% to 10.7% in laparoscopic low anterior resection in a retrospective study. The results of majority of the meta-analyses support the effectiveness of postoperative TDT placement in reducing the occurrence of AL (15–17).

Some studies (18, 19) have shown that the average time to confirm AL is approximately 7 postoperative days. This is because the anastomotic strength rapidly decreases in the early postoperative days until the fibroblasts and smooth muscle cells can synthesize large amounts of new collagen (20). However, we found that few studies contemplated the indwelling time of postoperative TDT placement, and reported that TDTs that were considered ineffective were often removed too early. For example, in the study by Zhao et al. (12) the indwelling time of TDT was only 4.2 days after surgery. In the studies of Chaline et al. (5) and Lee et al. (9), this time was only 4 and 3 postoperative days, respectively.

Hence, we performed a systematic review and meta-analysis of TDT and considered indwelling time as a critical factor to reanalyze the effectiveness of TDT placement in the prevention of AL after anterior rectal cancer surgery.

## Materials and methods

### Literature search

The meta-analysis was conducted in accordance with the PRISMA 2020 (21) and AMSTAR 2 (22) guidelines. A comprehensive literature search of PubMed, EMBASE, Cochrane Library, Clinicaltrials.gov, China Biology Medicine disc (CBMdisc), and China National Knowledge Infrastructure Whole Article Database (CNKI) databases were performed from inception to November 29, 2022 to determine the effect of all studies that compared the indwelling time of TDT on postoperative AL. All original research studies that compared the effectiveness of postoperative TDT placement and considered the indwelling time of TDT were included. The subject terms or keywords of the literature search were: “rectal cancer\tumor\neoplasm”, “anastomotic leaks\leakage” and “transanal drainage tube\catheter”. Original studies included in the relevant meta-analysis were also screened to identify other eligible studies.

### Inclusion and exclusion criteria

The inclusion criteria were: (1) original research studies that included patients with rectal cancer who underwent laparoscopic anterior resection; (2) randomized controlled trials (RCTs) or cohort studies, regardless of their publication status and languages; (3) studies that included patients with postoperative indwelling TDT; (4) studies which assessed the associations of TDT with AL and reoperation. Patients could be of any age, sex, country, and race. Moreover, patients who received preoperative neoadjuvant therapy and diverting stoma (DS) were also included.

The exclusion criteria were: (1) case-control studies, case reports, reviews, conference abstracts, and dissertations; (2) studies with duplicate data; (3) studies with insufficient data; (4) studies related to TDT used as the treatment for AL; and (5) studies related to anal balloon or anal stent.

### Data extraction and quality assessment

The title, abstract, and full text of the studies retrieved from the literature search were screened independently by two researchers. Any discrepancies were resolved through discussion with a senior professor. Two authors performed data extraction and collation separately, and any differences were resolved through dialogues until a consensus was reached or a third author was consulted. All extracted data were collated in an Excel spreadsheet. Data collected from the retrieved studies included publication journal, impact factor, first author’s name, whether TDT was effective based on the conclusion reached in the included study, publication year, study type, country, sample size, patient characteristics (age, sex, body mass index), DS, neoadjuvant therapy (radiochemotherapy or chemotherapy), AL and reoperation sample size, AL and reoperation rates, tube type, tube diameter, tube placement, tube position, decision to remove the TDT, the indwelling time of TDT, procedure type, stapling technique, tumor location, anastomosis location, and AL grading sample size (grade A+B, grade C). AL severity was graded according to the International Rectal Cancer Study Group (23), however, the sample of A- and B-grade AL were combined since grade A AL was rarely reported.

### Statistical analysis

All data preprocessing and analyses were conducted using R statistical software (R Foundation for Statistical Computing, Vienna, Austria). Publication bias was assessed by visual inspection of the funnel plot generated by the R software. Odds ratio (ORs) and 95% confidence intervals (CIs) were calculated for all dichotomous variables (Mantel–Haenszel statistical method). *I^2^* was used to assess the heterogeneity of the resulting evidence. If there was no evidence of heterogeneity, a fixed-effects model was used. Otherwise, a random-effects model was used. Since the heterogeneity among cohort studies was expected to be high due to their diversity, the random-effects meta-analysis approach was the default choice. Sensitivity analysis was performed if there were studies that significantly impacted the study heterogeneity. *p*-value for overall effect was calculated, and significance was set at *p* < 0.05.

### Subgroup analysis

The overall AL rate was the key metric. On this basis, subgroup analyses were performed, considering the indwelling time of TDT (≥ five days or <5 days). Time reported in the studies was used for time statistics if the specific indwelling time was reported. If time periods were reported, the median value was used. Based on the above subgroups, we focused on two additional indicators of DS and AL grading (A+B and C grades) to investigate the relationship between the indwelling time of TDT and both the abovementioned parameters.

## Results

### Literature search

**Figure 1:**
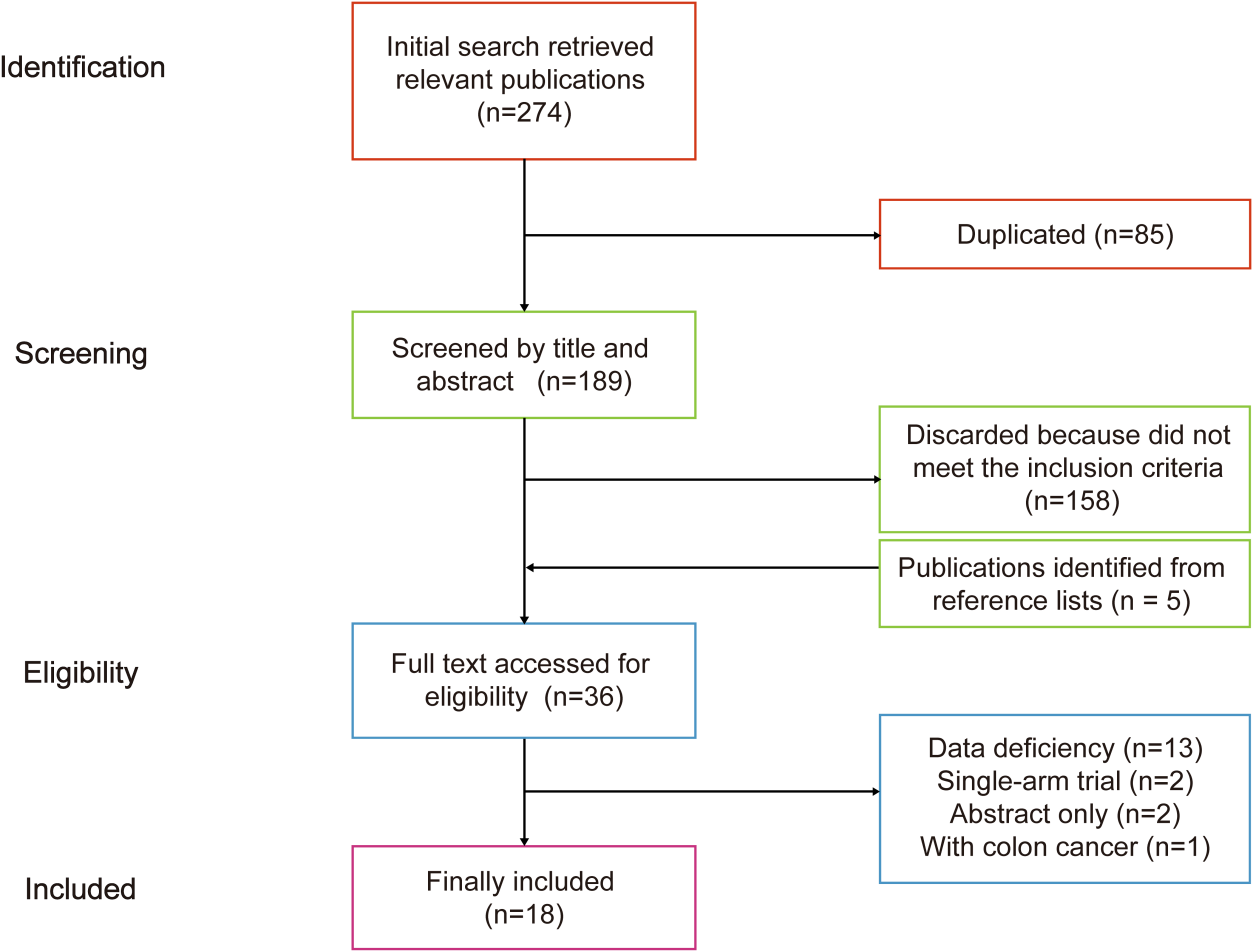
Flow chart of literature search.

A total of 274 relevant studies were identified in the initial search. Five additional studies were identified from the reference list of TDT-related meta-analysis (7–9, 14, 24). A total of 36 studies were subjected to full-text review. Eighteen publications were excluded: two were abstracts only, one was accompanied by colon cancer, two were single-arm trials, and 13 had insufficient clinical trials data. Therefore, a total of 18 studies met the inclusion criteria (**Figure 1**).

### Study characteristics

**Figure 2:**
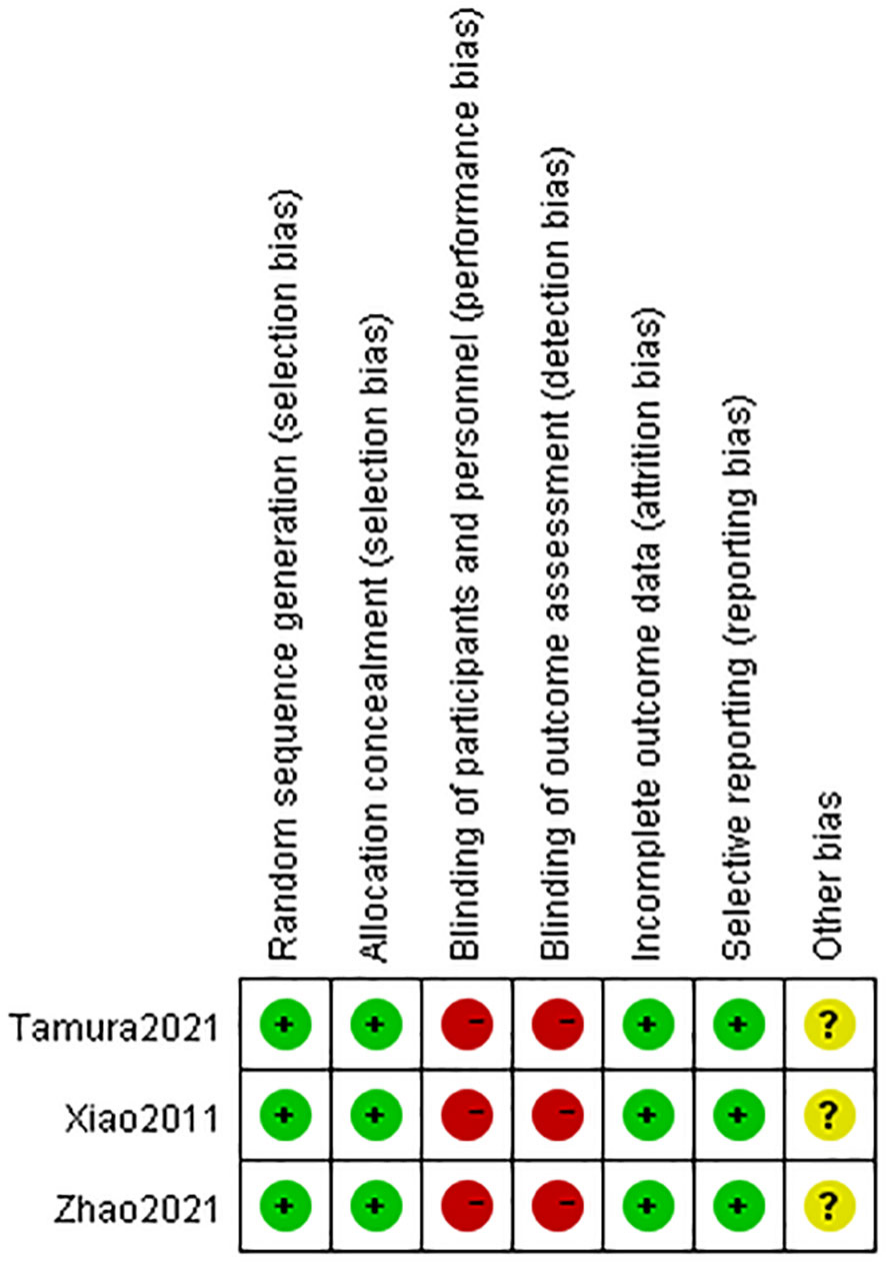
Meta-analysis results for whole studies of AL and reoperation.

Of all the included studies, three were RCTs (**Figure 2**) (10, 12, 25) and 15 were observational studies (prospective or retrospective studies) (4–9, 11, 13, 14, 24, 26–30). A total of 4805 patients were included in this meta-analysis, 2381 with TDT and 2494 without TDT. All studies reported the occurrence of AL; however, two RCTs (10, 12) did not report reoperation. The indwelling time of the TDT ranged between 3-7 days. A total of 14 studies (4, 6–8, 10, 11, 13, 14, 25–30) reported that the TDTs’ indwelling time was ≥5 days, suggesting that postoperative day 5 might be a critical time. Of the 18 included studies, only 6 reported on the time to confirm postoperative AL, and of these, 5 reported on the time to postoperative AL of 5 days or more - 5.8 d (27), 6.5 d (11), 6.8 d (24), and 10.1 d (13), respectively - and one of these reported on the division of AL into an early leakage group (POD ≤ 5, n = 9) and a late leakage group (POD ≥ 6, n = 16) (14). Therefore, we chose postoperative day 5 as the cutting point. As for the funded status of the included studies, three (11, 25, 27) of the included studies reported having scientific funding, the rest were non-profit, and none of the studies received funding from healthcare companies. The funded status of all included studies were collected in **Table 1B**. The essential characteristics of all the studies are summarized in **Tables 1**–**3**.

**Table 1A:** Surgical details of included studies.

Study	operation type	Type of drainage tube	diameter(mm)	Placement of drainage tube	Decision to remove	stapling technique	Subgroup	Indwelling time (days)	Anastomosis Position	
									TDT	Non-TDT
Kuk (27)	LAR, LLAR	silicone tube	10	Anal canal	no AL	Double-stapling technique	≥5	5 POD^a^	NR	NR
Zhao (21)	LLAR	silicone tube	9.3	5 cm above the anastomosis	No AL; early removal was allowed if the patient experienced intolerable pain	Double-stapling technique	< 5	4.2 POD^b^	4(3-5)^d^	4(3-5.5)^d^
Tamura (10)	LLAR	a Malecot latex tube	6.7-8	3-5 cm above the anastomotic line	no AL	Double-stapling technique	≥5	5 POD^a^	NR	NR
Li (30)	LAR	a 7.5# tracheal intubation	7.5	5 cm above the anastomotic line	fecal discharge or the passage of flatus was continuously observed	Double-stapling technique	≥5	5 POD^b^	3.95 ± 1.34 ^c^	4.6 ± 1.93 ^c^
Wang (4)	LLAR	a pleural drainage tube (36Fr)	12	the tip 30-50 mm proximal to the anastomotic site	NR	Double-stapling technique	≥5	7 POD^a^	4.5 ± 1.7 ^c^	5.5 ± 1.5 ^c^
Chaline (5)	LLAR	a Foley catheter	7.3	through the colorectal or coloanal anastomosis	NR	Double-stapling technique	<5	4 POD^b^	NR	NR
Carboni (7)	LAR, LLAR	Radiopaque soft silicone tube	20	Anal canal	no AL, passing faces and gas	Double-stapling technique OR hand-sewn	≥5	5 POD^a^	NR	NR
Kawada (14)	LLAR	Malecot tube	9.3	the tip 5 cm above the anastomotic site	NR	Double-stapling technique	≥5	5.5 POD^b^	NR	NR
Goto (26)	LAR\LLAR\RLAR	a silicone or rubber tube	10	3-5 cm above the anastomotic site	watery stool came out of the tube	double-stapling technique OR hand-sewn	≥5	5 POD^b^	5(3-7)^d^	7(5-10)^d^
Ito (29)	LLAR	Malecot tube	9.3	3 cm from the oral side of the anastomotic site	NR	Double-stapling technique	≥5	5 POD^a^	NR	NR
Yang (11)	LLAR, RLAR	rubber drainage tube	8/9.3	4 to 6 centimetres proximal to the anastomosis	faecal discharge or the passage of flatus was continuously observed	a circular stapler	≥5	5.3 POD^b^	4 ^e^	4.5 ^e^
Brandl (24)	LAR, LLAR	rubber latex foley catheter	9.3	the tip 5–10 cm proximal to the anastomosis	NR	Double-stapling technique OR hand-sewn	<5	4.9 POD^a^	NR	NR
Hidaka (6)	LLAR	Marecot catheter (28Fr) or pleats drain (10 mm)	9.3/10	the tip 30 mm proximal to the anastomotic site	NR	Double-stapling technique	≥5	7 POD^a^	4.5 ± 1.78 ^c^	5.09 ± 1.59 ^c^
Kim (8)	LAR, LLAR	silicone tube	7	above the anastomotic site	according to the patient’s general status and feeding process	Double-stapling technique	≥5	5 POD^b^	3.5 ± 1.8 ^c^	3.6 ± 1.5 ^c^
Lee (9)	LAR	rubber tube	3.3	the tip 5–10 cm proximal to the anastomosis	no AL	Double-stapling technique	<5	3 POD^a^	NR	NR
Nishigori (28)	LAR, LLAR	Ficon tube	8	3–5 cm from the oral side of the anastomosis	NR	Double-stapling technique	≥5	5 POD^a^	4.5 (2.0–6.5) ^d^	4.5 (1.0–7.5) ^d^
Zhao (13)	LAR	rubber drainage tube	8.7	e tip 3–5 cm proximal to the anastomotic site	fecal discharge and gas were continuously observed in transanal drainage fluid	single or double stapling technique	≥5	5.5 POD^b^	NR	NR
Xiao (25)	LAR	silicone tube	NR	Anal canal	NR	Double-stapling technique or Handsewn technique	≥5	6 POD^b^	NR	NR

AL, anastomotic leakage; LAR, low anterior resection; LLAR, laparoscopic low anterior resection; RLAR, robotic low anterior resection; POD, postoperative day; NR, not reported.

a Exact indwelling time of TDT reported in the studies.

b Values are median.

c Values are mean ± SD.

d Values are median (range).

e Values are exact numbers.

**Table 1B:** Funded status of the included studies.

Study	Financial support	Funding source
Kuk (27)	sponsored	The Soonchunhyang University Research Fund
Zhao (12)	non-profit	–
Tamura (10)	non-profit	–
Li (30)	non-profit	–
Wang (4)	non-profit	–
Chaline (5)	non-profit	–
Carboni (7)	non-profit	–
Kawada (14)	non-profit	–
Goto (26)	non-profit	–
Ito (29)	non-profit	–
Yang (11)	sponsored	The Kyungpook National University Research Fund
Brandl (24)	non-profit	–
Hidaka (6)	non-profit	–
Kim (8)	non-profit	–
Lee (9)	non-profit	–
Nishigori (28)	non-profit	–
Zhao (13)	non-profit	–
Xiao (25)	sponsored	TheGuiding Scientific Project of Social Development from Zhenjiang

**Table 2A:** Main characteristics of included studies.

Study	Journal	Conclusion	Study type	Nationality	Sample (n)			Gender (M/F, n)		Age (years)	
					Total	TDT	Non-TDT	TDT	Non-TDT	TDT	Non-TDT
Kuk (27)	Asian J Surg	Yes	R,CS	Korea	556	215	341	140/75	200/141	61.6 ± 12.1^a^ (62,33-81)^b^	61.1 ± 11.3 ^a^ (61,36-90) ^b^
Zhao (12)	JAMA Surg	No	RCT	China	560	280	280	177/103	169/111	61.5 (54.0-68.8) ^b^	62.0 (52.0-69.0) ^b^
Tamura (10)	Am J Surg	No	RCT	Japan	157	79	78	51/28	50/28	69 [40-90] ^b^	69[39-91] ^b^
Li (30)	J BUON	Yes	R,CS	China	222	86	136	52/34	75/61	41.9%≥60^c^	42.6%≥60 ^c^
Wang (4)	Asian Pac J Cancer Prev	Yes	R,CS	China	220	120	100	70/50	64/36	58.6 ± 11.3 ^a^	57.3 ± 10.1 ^a^
Chaline (5)	Tech Coloproctol	No	R,CS	France	144	72	72	51/21	51/21	64.0 ± 12.2 ^a^	60.2 ± 12.0 ^a^
Carboni (7)	Transl Gastroenterol Hepatol	No	PSM	Italy	429	275	154	146/129	85/69	61.1 [19–83] ^b^	60.6 [40–79] ^b^
Kawada (14)	Int J Colorectal Dis	Yes	R,CS	Japan	201	178	23	141/60		66 (28–89) ^b^	
Goto (26)	J Surg Oncol	Yes	R,CS	Japan	328	205	123	143/62	77/46	67 (60-74) ^b^	70 (62-77) ^b^
Ito (29)	Asian J Endosc Surg	Yes	R,CS	Japan	69	28	41	21/7	27/14	69.7 ± 7.4 ^a^	66.4 ± 11.4 ^a^
Yang (11)	Colorectal Dis	No	R,CS	Korea	204	102	102	65/37	66/36	64.2^d^	63.5 ^d^
Brandl (24)	Ann Med Surg (Lond)	Yes	R,CS	Austria/Germany	242	139	103	81/58	58/45	63.2 ± 13.4 ^a^	63.9 ± 15.5 ^a^
Hidaka (6)	Surg Endosc	Yes	R,CS	Japan	205	96	109	64/32	65/44	63.4 ± 10.2 ^a^	64.6 ± 12.3 ^a^
Kim (8)	Ann Surg Treat Res	No	R,CS	Korea	70	35	35	21/14	23/12	62.2 ± 11.1 ^a^	59.3 ± 10.7 ^a^
Lee (9)	Langenbecks Arch Surg	No	PSM	Korea	536	154	382	103/51	245/137	63.6 ± 11.3 ^a^	68.2 ± 10.6 ^a^
Nishigori (28)	World J Surg	Yes	R,CS	Japan	176	36	140	23/13	88/52	61 (35–75) ^b^	63 (30–88) ^b^
Zhao (13)	World J Surg	No	nonrandomized prospective study	China	158	81	77	47/34	43/34	37%≥60 ^c^	46.8%≥60 ^c^
Xiao (25)	World J Surg	Yes	RCT	China	398	200	198	115/85	121/77	59 ± 11 ^a^	58 ± 12 ^a^

RCT, randomized controlled trial; R, retrospective; CS, cohort study; PSM, propensity score matching; BMI, body mass index; NR, not reported; TDT, transanal drainage tube.

a Values are mean ± SD.

b Values are median (range).

c Values are percentage.

d Values are median.

**Table 2B:** Main characteristics of included studies.

Study	BMI (kg/m2)		Diverting stoma (n)		Preoperative neoadjuvant therapy (n)		Tumor location	
	TDT	Non-TDT	TDT	Non-TDT	TDT	Non-TDT	TDT	Non-TDT
Kuk (27)	23.7 ± 3.0 ^a^ (23.4, 16.7-26.8) ^b^	23.5 ± 3.1^a^ (23.7,17.9-31.8) ^b^	0	0	28	61	54/90/71(Lower/Middle/Upper) ^e^	72/120/149(Lower/Middle/Upper) ^e^
Zhao (12)	23.1 (20.8-24.6) ^b^	22.8 (20.7-25.2) ^b^	72	89	12	16	7.1 ± 1.9 ^a^	7.2 ± 2.1 ^a^
Tamura (10)	39.2%≥22.1 ^c^	48.7%≥22.1 ^c^	34	37	10	19	27/29/23(Lower/Middle/Upper) ^e^	21/27/30(Lower/Middle/Upper) ^e^
Li (30)	21.18 ± 2.09 ^a^	20.99 ± 2.17 ^a^	0	0	0	0	30/56(Lower/Middle/Upper) ^e^	48/88(Lower/Middle/Upper) ^e^
Wang (4)	25.8 ± 6.1 ^a^	24.7 ± 5.3 ^a^	0	0	0	0	6.8 ± 2.0 ^a^	9.1 ± 2.9 ^a^
Chaline (5)	25.5 ± 4.4 ^a^	25.1 ± 4.6 ^a^	72	72	41	47	29/17/26(Lower/Middle/Upper) ^e^	26/20/26(Lower/Middle/Upper) ^e^
Carboni (7)	NR	NR	0	54	110	59	75/105/95(Lower/Middle/Upper) ^e^	30/63/61(Lower/Middle/Upper) ^e^
Kawada (14)	22.0(10.5-32.6) ^b^		0	0	29		63/138(Lower/Middle) ^e^	
Goto (26)	22 (20.2-24.1) ^b^	22.7 (20.2-25.2) ^b^	8	19	30	6	8 (6–10) ^b^	8 (6-10) ^b^
Ito (29)	NR	NR	12	14	0	0	24/28/17(Rb, Ra, Rs) ^e^	
Yang (11)	23.9 (16.9-31.7) ^b^	23.5 (16.6-33.2) ^b^	0	0	24	21	14/72/16(Lower/Middle/Upper) ^e^	17/69/16(Lower/Middle/Upper) ^e^
Brandl (24)	NR	NR	41	22	0	0	72/67(Lower/Middle) ^e^	47/56(Lower/Middle) ^e^
Hidaka (6)	21.2 ± 3.2 ^a^	21.8 ± 3.4 ^a^	0	0	0	0	54/42(Ra, Rb) ^e^	87/22(Ra, Rb) ^e^
Kim (8)	23.7 ± 2.5 ^a^	22.7 ± 2.9 ^a^	0	0	0	0	8.8 ± 1.2 ^a^	8.9 ± 1.4 ^a^
Lee (9)	24.1 ± 3.6 ^a^	23.8 ± 4.6 ^a^	0	0	19	58	70/84(Lower/Middle/Upper) ^e^	169/213(Lower/Middle/Upper) ^e^
Nishigori (28)	22.0 (15.0–28.0) ^b^	22.4 (15.3–31.2) ^b^	0	0	0	1	21/15(Ra, Rb) ^e^	94/46(Ra, Rb) ^e^
Zhao (13)	NR	NR	0	0	0	0	22/46/13(Lower/Middle/Upper) ^e^	21/38/18(Lower/Middle/Upper) ^e^
Xiao (25)	24.2 ± 3.8 ^a^	23.7 ± 4.0 ^a^	0	0	0	0	7 (3.5–11) ^b^	8 (3.5–11) ^b^

RCT, randomized controlled trial; R, retrospective; CS, cohort study; PSM, propensity score matching; BMI, body mass index; NR, not reported; TDT, transanal drainage tube; Rs, rectosigmoid; Ra, rectum above the peritoneal reflection; Rb, rectum below the peritoneal reflection.

a Values are mean ± SD.

b Values are median (range).

c Values are percentage.

d Values are median.

e Values are numbers.

**Table 3:** Risk of bias summary for all included cohort studies.

Study	Confunding	Selection	Classification	Deviations from intended interventions	Missing data	Measurement of outcomes	Selection of the reported result	Overall
Kuk (27)	Moderate	Moderate	NR	Low	Low	Low	Moderate	Moderate
Li (30)	Moderate	Moderate	NR	Low	Low	Low	Moderate	Moderate
Wang (4)	Moderate	Moderate	NR	Low	Low	Low	Moderate	Moderate
Chaline (5)	Low	Moderate	NR	Low	Low	Low	Moderate	Low
Carboni (7)	Low	Moderate	NR	Low	Low	Low	Moderate	Low
Kawada (14)	Moderate	Moderate	NR	Low	Low	Low	Moderate	Moderate
Goto (26)	Moderate	Moderate	NR	Low	Low	Low	Moderate	Moderate
Ito (29)	Moderate	Moderate	NR	Low	Low	Low	Moderate	Moderate
Yang (11)	Low	Moderate	NR	Low	Low	Low	Moderate	Low
Brandl (24)	Moderate	Moderate	NR	Low	Low	Low	Moderate	Moderate
Hidaka (6)	Moderate	Moderate	NR	Low	Low	Low	Moderate	Moderate
Kim (8)	Moderate	Moderate	NR	Low	Low	Low	Moderate	Moderate
Lee (9)	Low	Moderate	NR	Low	Low	Low	Moderate	Low
Nishigori (28)	Moderate	Moderate	NR	Low	Low	Low	Moderate	Moderate
Zhao (13)	Low	Moderate	NR	Low	Low	Low	Moderate	Low

NR, not reported.

### Results of the meta-analysis 

**Figure 3:**
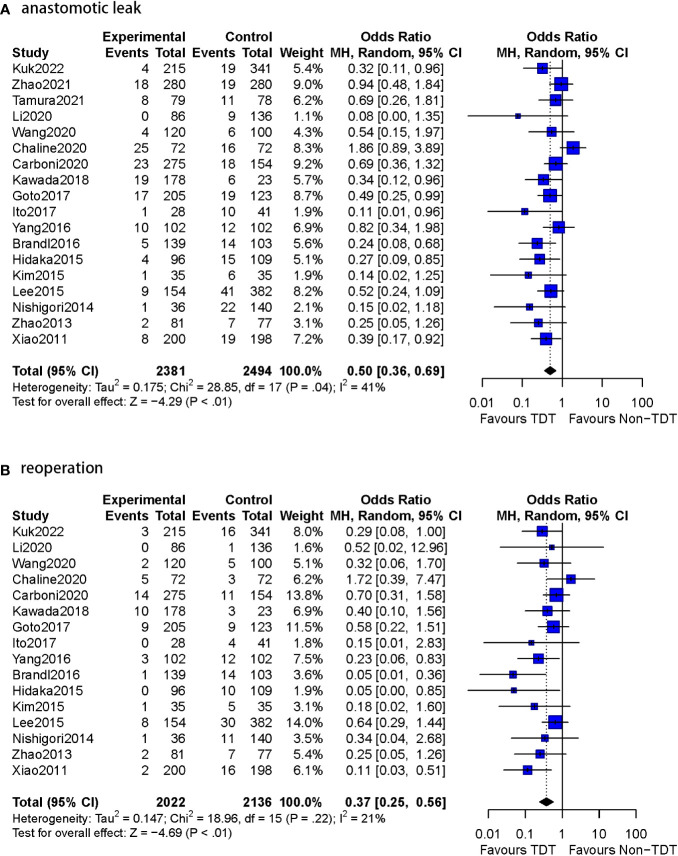
Meta-analysis results for whole studies of AL and reoperation. **(A)**: anastomotic leak. **(B)**: reoperation.

The effectiveness of TDT placement after anterior resection of rectal cancer is controversial (5, 7–13). However, most of the included studies (4, 6, 14, 24–30) confirmed that postoperative TDT placement was effective in reducing the occurrence of AL and reoperation (**Table 4**, **Figure 3**).

**Table 4:** The main results of AL and reoperation of the included studies.

studies	AL(n)		reoperation(n)		AL (grade A+B) (n)		AL (grade C) (n)		AL rate(%)		Reoperation rate(%)		AL rate (grade A+B) (%)		AL rate (grade C) (%)	
	TDT	Non-TDT	TDT	Non-TDT	TDT	Non-TDT	TDT	Non-TDT	TDT	Non-TDT	TDT	Non-TDT	TDT	Non-TDT	TDT	Non-TDT
Kuk (27)	4	19	3	16	1	3	3	16	1.9	5.6	1.4	4.7	0.5	0.9	1.4	4.7
Zhao (12)	18	19	NR	NR	14	11	4	8	6.4	6.8	NR	NR	0.1	3.9	1.4	2.9
Tamura (10)	8	11	NR	NR	5	9	1	1	10.1	14.1	NR	NR	6.3	11.5	1.3	1.3
Li (30)	0	9	0	1	0	8	0	1	0	6.6	0	0.7	0	5.9	0	0.7
Wang (4)	4	6	2	5	2	1	2	5	3.3	6	1.7	5	1.7	1	1.7	5
Chaline (5)	25	16	5	3	25	16	4	2	34.7	22.2	6.9	4.2	34.7	22.2	5.6	2.8
Carboni (7)	23	18	14	11	9	7	14	11	8.4	11.7	5.1	7.1	3.3	4.5	5.1	7.1
Kawada (14)	19	6	10	3	9	3	10	3	10.7	26.1	5.6	13	5.1	13	5.6	13
Goto (26)	17	19	9	9	10	11	7	8	8.3	15	4.4	7.3	4.9	8.9	3.4	6.5
Ito (29)	1	10	0	4	1	6	0	4	3.6	24.4	0	9.8	3.5	14.6	0	9.8
Yang (11)	10	12	3	12	7	0	3	12	9.8	11.8	2.9	11.8	6.9	0	2.9	11.8
Brandl (24)	5	14	1	14	4	0	1	14	3.6	13.4	0.7	13.4	2.9	0	0.7	13.6
Hidaka (6)	4	15	0	10	4	5	0	10	4.2	13.8	0	9.2	4.1	4.6	0	9.2
Kim (8)	1	6	1	5	0	1	1	5	2.9	17.1	2.9	14.3	0	2.9	2.9	14.3
Lee (9)	9	41	8	30	1	11	8	30	5.8	10.7	5.2	7.9	0.6	2.9	5.2	7.9
Nishigori (28)	1	22	1	11	0	11	1	11	2.7	15.7	2.7	7.8	0	7.9	2.8	7.9
Zhao (13)	2	7	2	7	0	0	2	7	2.5	7.8	2.5	7.8	0	0	2.5	9.1
Xiao (25)	8	19	2	16	6	3	2	16	4	9.6	1	8.1	3	1.5	1	8.1

AL, anastomotic leakage; TDT, transanal drainage tube; NR, not reported.

### Results of subgroup analysis

**1. TDT indwelling time ≥ 5 days group (14 studies)**

1.1 AL rate

**Figure 4:**
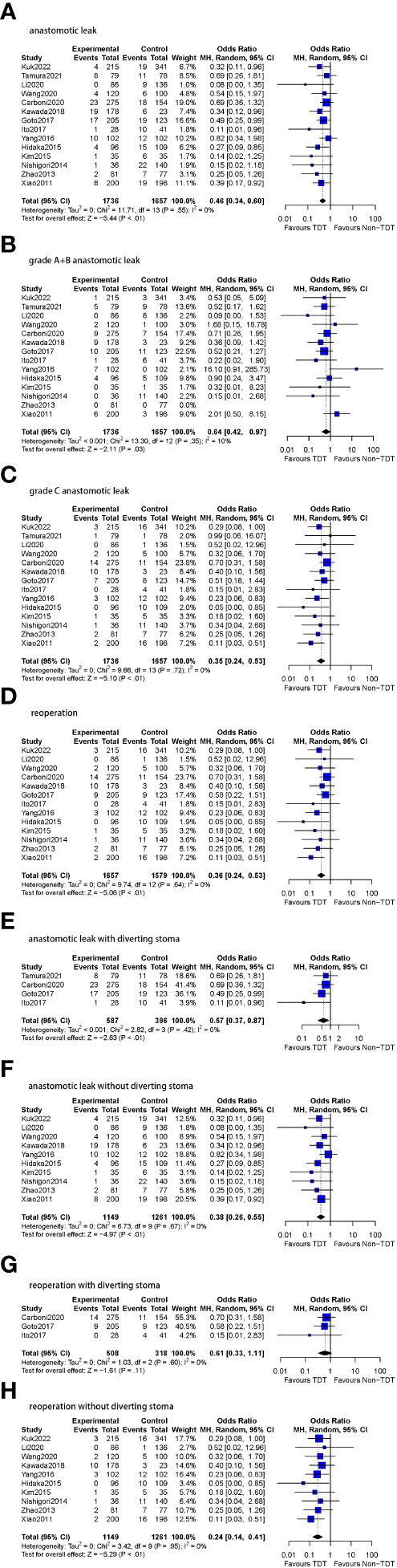
Meta-analysis results for TDT indwelling time ≥5 days subgroup. **(A)**: anastomotic leak. **(B)**: grade A+B anastomotic leak. **(C)**: grade C anastomotic leak. **(D)**: reoperation. **(E)**: anastomotic leak with diverting stoma. **(F)**: anastomotic leak without diverting stoma. **(G)**: reoperation with diverting stoma. **(H)**: reoperation without diverting stoma.

The postoperative AL rate was used as an outcome indicator in all 14 studies (n=3393 patients). A total of 5.9% (102/1736) patients with TDT were reported to have AL compared to 10.8% (179/1657) in the non-TDT group (OR=0.46, 95%CI 0.34-0.60, *p*<0.01). No heterogeneity was observed in this analysis (I^2^ = 0%, *p* = 0.55) (**Figure 4**).

1.1.1 AL rate (grade A+B)

All 14 studies included in this subgroup reported A and B-level AL (n=3393 patients). A total of 3.1% (54/1736) patients were assessed to have grade A or BAL compared to 4.1% (68/1657) patients following no TDT (OR=0.64, 95%CI 0.42-0.97, *p*=0.03). Low heterogeneity was found in this analysis (I^2^ = 10%, *p* = 0.35).

1.1.2 AL rate (grade C)

All 14 studies included in this subgroup reported C-level AL (n=3393 patients). In patients with a TDT placed for ≥5 days, 2.6% (46/1736) patients were diagnosed with grade CAL compared to 6.0% (100/1657) patients following no TDT (OR=0.35, 95%CI 0.24-0.53, *p*<0.01). No heterogeneity was found in this analysis (I^2^ = 0%, *p* = 0.72).

1.2 Reoperation rate

Thirteen of the 14 studies in this subgroup reported reoperation rates (n=3236 patients). The specific number of reoperation cases was not reported in two studies (10, 12). Reoperations were reported in 2.8% (47/1657) of the patients with TDT compared to 7.0% (110/1579) patients without TDT (OR=0.36, 95%CI 0.24-0.53, *p*<0.01). No heterogeneity was observed (I^2^ = 0%, *p* = 0.64).

1.3 DS

Of the 14 studies included in the subgroup analysis, four (n=983 patients) reported DS and 10 (n=2410 patients) did not.

1.3.1 AL

In the patients who with prophylactic DS, AL was reported in a total of 8.3% (49/587) patients with TDT in comparison to 14.6% (58/396) patients without TDT (OR=0.57, 95%CI 0.37-0.87, *p*<0.01), and no heterogeneity was found (I^2^ = 0%, *p* = 0.42). In the patients who without prophylactic DS, AL was reported in 9.6% (121/1261) patients without TDT compared to 4.6% (53/1149) patients with TDT (OR=0.38, 95%CI 0.26-0.55, *p*<0.01), and no heterogeneity was found as well (I^2^ = 0%, *p* = 0.67).

1.3.2 Reoperation rate

Three of the four studies reported the reoperation rate of patients with DS (n=826 patients). Reoperations were performed in 4.5% (23/508) patients with TDT and in 7.5% (24/318) patients without TDT (OR=0.61, 95%CI 0.33-1.11, *p*=0.11), and no heterogeneity was found (I^2^ = 0%, *p* = 0.60). All 10 studies reported reoperation rates of patients without DS (n=2410 patients). 2.1% (24/1149) patients with TDT underwent reoperations in comparison to 6.8% (86/1261) patients without TDT (OR=0.24, 95%CI 0.14-0.41, *p*<0.01). Similarly, no heterogeneity was observed (I^2^ = 0%, *p* = 0.95).

**2. TDT indwelling time < 5 days group (4 studies)**

2.1 AL rate

**Figure 5:**
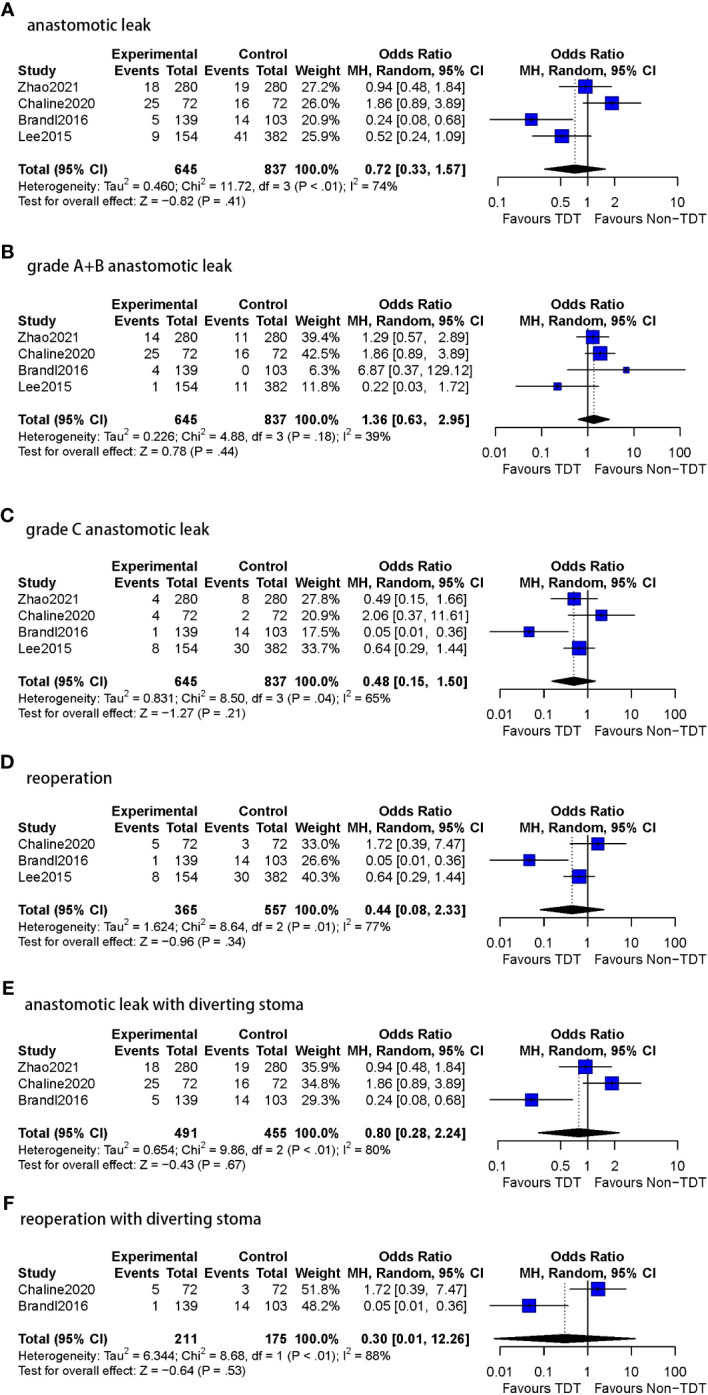
Meta-analysis results for TDT indwelling time less than 5 days subgroup. **(A)**: anastomotic leak. **(B)**: grade A+B anastomotic leak. **(C)**: grade C anastomotic leak. **(D)**: Reoperation. **(E)**: anastomotic leak with diverting stoma. **(F)**: reoperation with diverting stoma.

Postoperative AL rate was reported in all four studies in which the TDT indwelling time was <5 days (n=1482 patients). 8.8% (57/645) patients with TDT were reported to have AL compared to 10.8% (90/837) in the non-TDT group, with a pooled OR of 0.72 (95%CI 0.33-1.57, *p*=0.41). Substantial heterogeneity was observed in this analysis (I^2^ = 74%, *p*<0.01) (**Figure 5**).

2.1.1 AL rate (grade A+B)

All four studies included in this subgroup reported A and B grades of AL (n=1482 patients). Among patients with a TDT placed for <5 days, 6.8% (44/645) were assessed to have grade A or BAL compared to 4.5% (38/837) following no TDT (OR=1.36, 95%CI 0.63-2.95, *p*=0.44). Moderate heterogeneity was observed (I^2^ = 39%, *p* = 0.18).

2.1.2 AL rate (grade C)

All four studies included in this subgroup reported grade C AL (n=1482 patients). In patients with a TDT placed for <5 days, 2.6% (17/645) were diagnosed with grade CAL compared to 6.5% (54/837) with no TDT (OR=0.48, 95%CI 0.15-1.50, *p*=0.21). Moderate heterogeneity was observed (I^2^ = 65%, *p* = 0.04).

2.2 Reoperation rate

Three of the four studies in this subgroup reported reoperation rates (n=922 patients). Reoperations were reported in 3.8% (14/365) patients with TDT in comparison to 8.4% (47/557) patients without TDT (OR=0.44, 95%CI 0.08-2.33, *p*=0.34). Substantial heterogeneity was observed in this analysis (I^2^ = 77%, *p* = 0.01).

2.3 DS

In this subgroup, three studies (n=946 patients) reported DS and only one study (n=536 patients) did not.

2.3.1 AL rate

Three of the four studies reported DS in the less than 5 days TDT indwelling time subgroup. AL was reported in 9.8% (48/491) patients with TDT in comparison to 10.8% (49/455) patients without TDT (OR=0.80, 95%CI 0.28-2.24, *p*=0.67), and substantial heterogeneity was observed (I^2^ = 80%, *p*<0.01).

Only one study in this subgroup did not report DS. AL was reported in 5.8% (9/154) patients with TDT compared to 10.7% (41/382) patients without TDT (OR=0.52, 95%CI 0.24-1.09, *p*=0.08).

2.3.2 Reoperation rate

In the two of three studies reporting DS in the less than 5 days TDT indwelling time subgroup (n=386 patients), reoperations were performed in 2.8% (6/211) patients with TDT and in 9.7% (17/175) patients without TDT (OR=0.30, 95%CI 0.01-12.26, *p*=0.53), substantial heterogeneity was found as well (I^2^ = 88%, *p*<0.01).

Only one study reported the reoperation rate in patients without DS (n=536). 5.2% (8/154) patients with TDT underwent reoperations compared to 7.9% (30/382) without TDT (OR=0.64, 95%CI 0.29-1.44, *p*=0.28).

### Sensitivity analysis

Substantial heterogeneity was found in the subgroup of TDT indwelling time of < 5 days. In the AL indicator, heterogeneity was slightly reduced after individual study (5) was excluded; However, substantial heterogeneity remained (*I* ^2^ = 59%, *p*=0.09). Heterogeneity was reduced to 24% after individual study (24) was excluded from the meta-analysis of reoperations in the subgroup (*p*=0.73). Guo et al. (15) concluded that in the study by Challine et al. (5), temporary diverting stoma were systemically constructed for all patients after anterior resection for rectal cancer, which may have contributed to the differences from other studies in the pooled analysis.

### Publication bias

**Figure 6:**
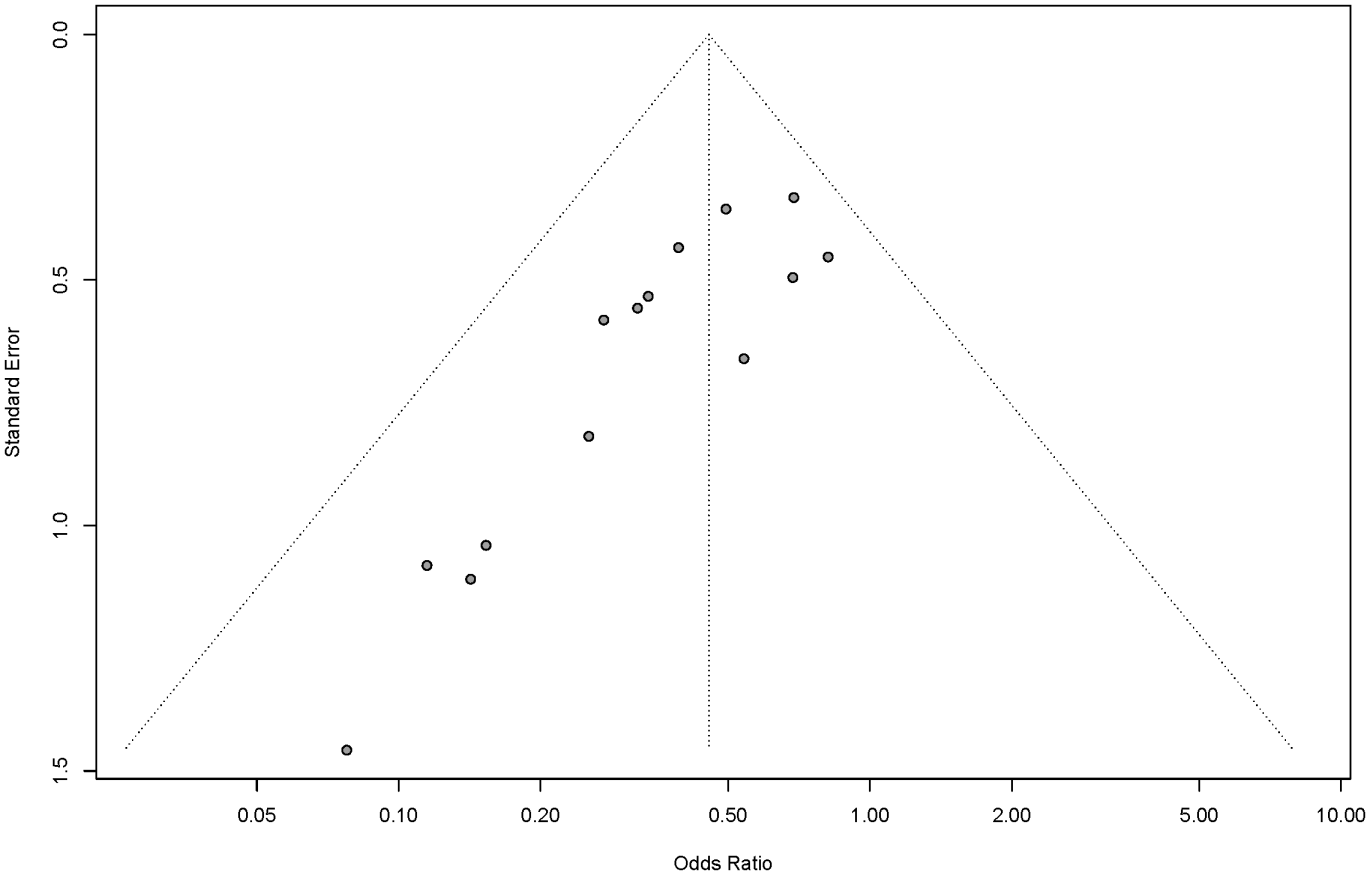
Funnel plot of ≥5-days subgroup.

According to the Egger test, there was evidence of publication bias in the meta-analysis of AL (*p*=0.0005), reoperation (*p*=0.0114), and AL without DS (*p*=0.0032) in the subgroup with TDT indwelling time ≥5 days (**Figure 6**).

## Discussion

We conducted a systematic review and meta-analysis of the indwelling time of TDT after anterior rectal cancer surgery. After conducting a systematic analysis of the preliminary data statistics, we finalized the subgroups as ≥5-day and <5-day subgroups depending on the indwelling time of the TDT postoperatively. The vast majority of the 18 included studies (14/18) had TDT indwelling time of 5 days or more, which is also consistent with our clinical experience. The results showed that the indwelling time of TDT for ≥5-days was associated with a significant reduction (TDT vs. Non-TDT) in overall AL, grade A+B AL, grade C AL, overall reoperation rate and that in patients without a prophylactic DS. There were no statistically significant differences in any of the abovementioned indicators when the indwelling time of TDT was less than 5 days.

In most of the included studies, TDT was maintained until at least postoperative day (POD) 5. This may differ from what most surgeons believe, that postoperative AL does not occur only in the early postoperative period. Several studies have reported that the mean time to postoperative AL was ≥5 days, and that most occurred on POD 7 (18, 19, 31). Li et al. (19) defined AL occurring ≤ 5 days postoperatively as very early AL (vE-AL). AL occurring during this period is considered fatal; thus, POD 5 was considered the optimal cutoff time to distinguish truly life-threatening early AL requiring urgent reoperation. Several factors may have contributed to these findings. First, during the early postoperative period, the anal sphincter is usually tight due to factors such as pain, tension, or inflammation, which may lead to increased pressure in the rectal lumen as stool or gas passes through the anastomosis, thus interfering with anastomosis healing (4). Kwada et al. reported that the daily fecal volume increased until POD 3 or 4 and significantly decreased on POD 5 (14), which means that the pressure in the rectal lumen increases until POD 5, leading to an increased risk of AL. Second, the strength of the anastomosis is mainly dependent on the collagen fibrils within the submucosal layer. During the first few days after rectal surgery, collagen at the anastomosis degrades, and the strength of the anastomosis depends on the suture- or staple-holding capacity of the existing collagen. AL is more likely to occur at this stage until a large amount of collagen can be resynthesized within one or two postoperative days. AL occurs when the radial force at the anastomosis exceeds the resistance generated by sutures, staples, and early scars. Bursting pressure approaches 100% on POD 7, after which the intestine generally bursts outside the anastomotic site (20). In colon, the strength at the anastomosis site has been reported to reach only 30% of the initial strength after 48 h of surgery (32) and reach 50% after one week (20). These findings suggest that premature TDT removal may be detrimental to postoperative anastomotic healing.

We analyzed the number and rates of occurrence of grade A+B and grade C of AL in the included studies. Some of the included studies did not report accurate data on AL classification, so patients with grade C AL were considered as those requiring reoperation for statistical purposes. The results may not be accurate because patients with AL, not only grade C AL, can be treated surgically.

In clinical practice, surgeons often use a prophylactic DS to reduce the occurrence of AL in patients at high risk for postoperative AL. Preventive ileostomy can reduce the passage of stool and gas through the anastomosis and reduce the pressure in the colorectal lumen to prevent AL (4). In particular, prophylactic ileostomy can reduce the incidence of clinically significant AL, resulting in lower reoperation rates in rectal cancer (33). However, preventive ileostomy has disadvantages such as inconvenience, high incidence of stoma complications, poor patient subjective perception, and the need for a secondary surgery to close the stoma (34), which increases the hospital and surgical costs and produces a secondary trauma to the patient. TDT can also reduce postoperative pressure in the rectal lumen and be an alternative to preventive ileostomy in patients with a clear low risk of postoperative AL (35). The effect of DS on AL is not negligible. We also created statistics for this purpose. And our findings suggest that different stoma status does not impact the effect of TDT at a specific indwelling time (**Table 5**).

**Table 5:** Results of subgroup with indicator of diverting stoma.

Subgroup	indicator		TDT	Non-TDT	*p*
≥5 days	AL rate (%)	DS	8.3	14.6	<0.01
		Non-DS	4.6	9.6	<0.01
	Reoperation rate (%)	DS	4.5	7.5	0.11
		Non-DS	2.1	6.8	<0.01
<5 days	AL rate (%)	DS	9.8	10.8	0.67
		Non-DS	5.8	10.7	0.08
	Reoperation rate (%)	DS	2.8	9.7	0.53
		Non-DS	5.2	7.9	0.28

AL, anastomotic leakage; DS, diverting stoma.

Tumor location has now been identified as an independent important risk factor for postoperative AL (36). Tumors less than 5 cm from the anal verge are 6.5 times more likely to develop AL after surgery compared to those situated greater than 5 cm from the anal verge. We aimed to investigate the relationship between anastomotic position and AL after rectal cancer surgery at specific TDT indwelling times. This may be a very important factor affecting AL after rectal cancer surgery. Since anastomotic positions were not reported in some of the included studies, tumor positions were counted as the replacement of the anastomotic positions. However, the data on tumor locations reported in the included studies were inaccurate and in different ways, which did not allow valid grouping; therefore, no further analysis was performed. The relevant data reported in the included studies were organized in the **Tables 2**, **1**.

For unresectable rectal cancer, Guadagni et al. (37) mention that the integration of hypoxic pelvic perfusion (HPP)/targeted therapies may be effective in terms of locally controlled symptom as well as long term outcomes in patients with unresectable rectal cancer. This may provide an important adjunct to surgery for rectal tumors that are difficult to resect, and pelvic perfusion therapy may allow for more complete local tumor clearance, which in turn reduces anastomotic tumor recurrence and AL.

The concept of enhanced recovery after surgery (ERAS) has been generally accepted in rectal cancer surgery, with the advantage of reduced length of hospital stay (LOS), lower costs, and decreased non-surgical complications (38). Regrettably, none of the studies included in the present meta-analysis referred to ERAS protocols, which may be a limitation of this study. However, we assumed that the presence of TDT may not be in conflict with early postoperative feeding and ERAS protocols. Early postoperative feeding usually begins with liquid diet which produces minimal amount of loose stool and is unlikely to obstruct to the TDT. An indwelling TDT can drain stools and gas out of the rectal lumen, decreasing the intraluminal-pressure, providing an ideal environment for anastomotic healing, and eventually reducing the risk of AL. To some extent, postoperative indwelling TDT is beneficial for patients and may represent a new part of ERAS protocols.

This is the first meta-analysis to analyze the effect of indwelling time of TDT on the effectiveness of TDT after rectal cancer anterior resection. However, this study also had some limitations. The inclusion of less number of RCTs may have affected the statistical power and reduced the persuasiveness of the results. In terms of subgroups, there were fewer studies in the <5-day subgroup, which may have led to selection bias, and substantial heterogeneity was found in this subgroup. In addition, the included studies differed in the type and diameter of TDT, which may have introduced heterogeneity into the results. We assume that in addition to indwelling time, factors such as placement of the TDT catheter tip (proximal to the anastomosis or at the anastomosis), caliber of the TDT, material, and number of drainage holes may also affect AL. Further studies are needed to investigate this aspect.

## Conclusions

Extending the postoperative indwelling time of TDT to 5 days may reduce the overall AL and the need for reoperation in patients without a prophylactic DS.

## Data availability statement

The original contributions presented in the study are included in the article/supplementary material. Further inquiries can be directed to the corresponding author.

## Author contributions

XX: Data curation, Formal analysis, Writing – original draft. XZ: Conceptualization, Writing – review & editing. XL: Writing – original draft. AY: Data curation, Writing – original draft. XQZ: Data curation, Writing – original draft. SD: Writing – review & editing. ZL: Writing – review & editing, Formal analysis. ZC:Writing – original draft. KW: Supervision, Writing – review & editing.
